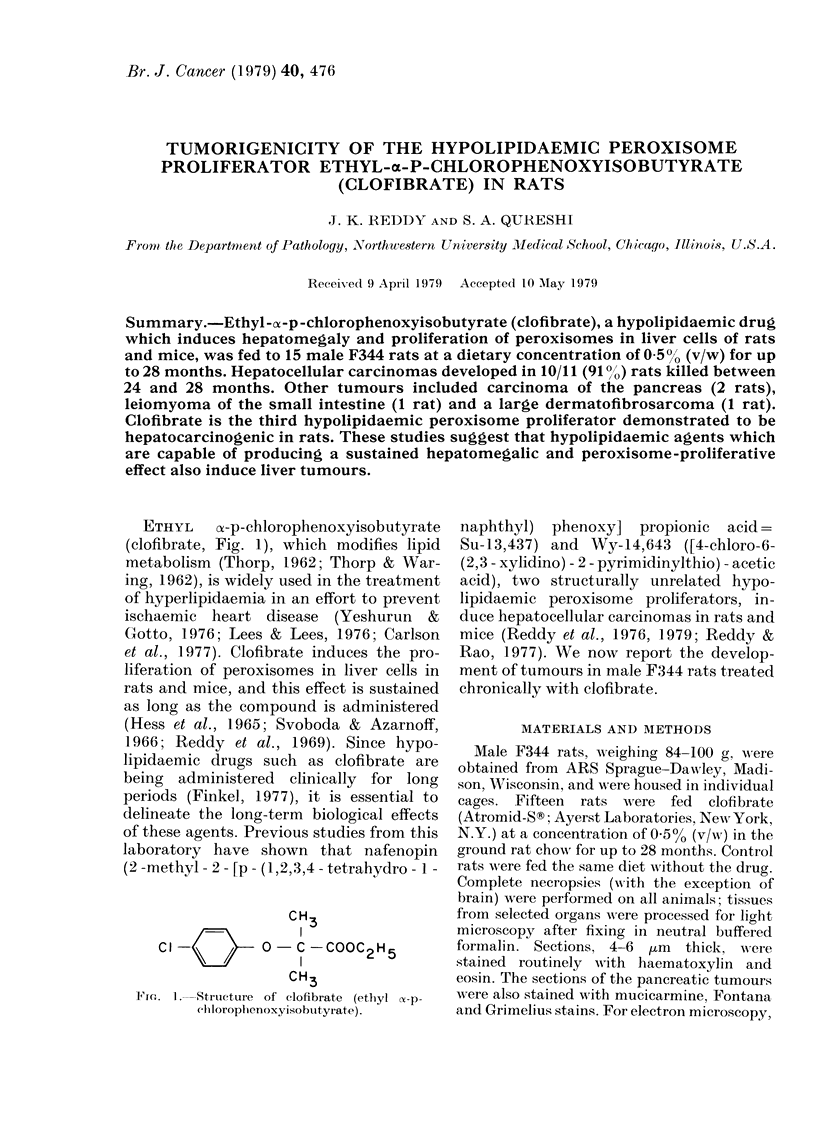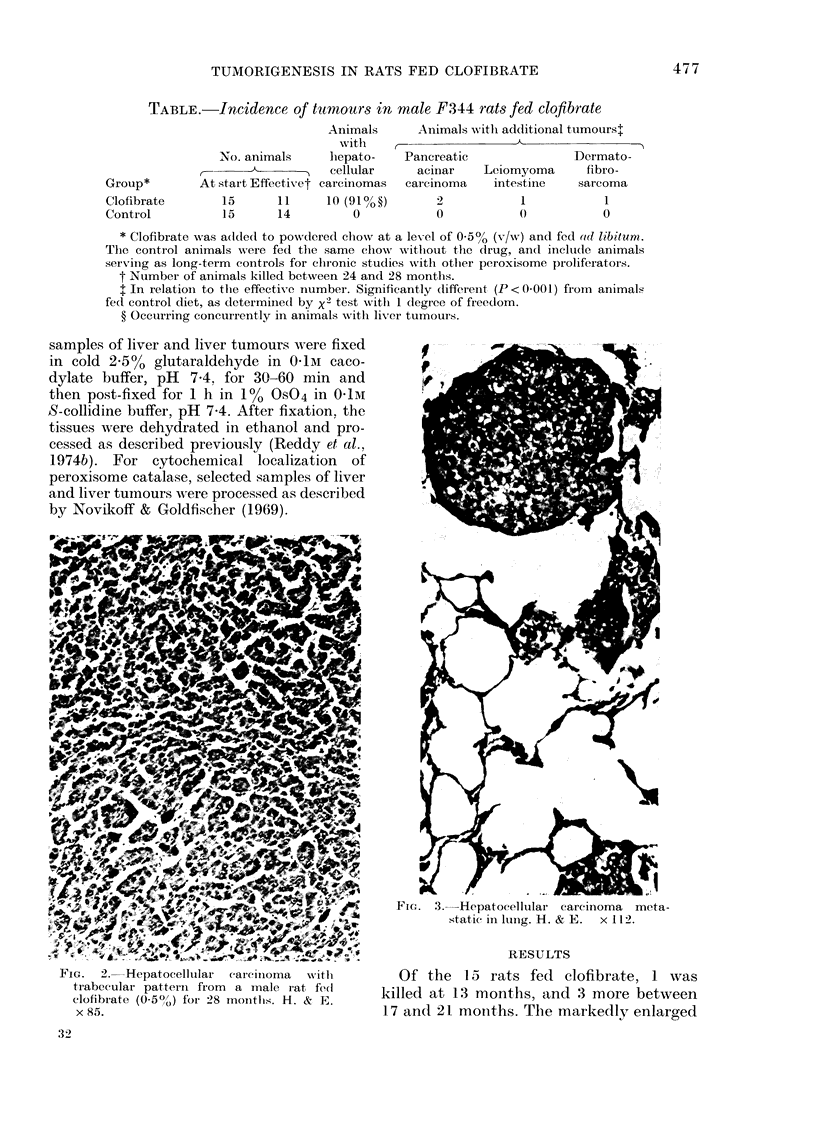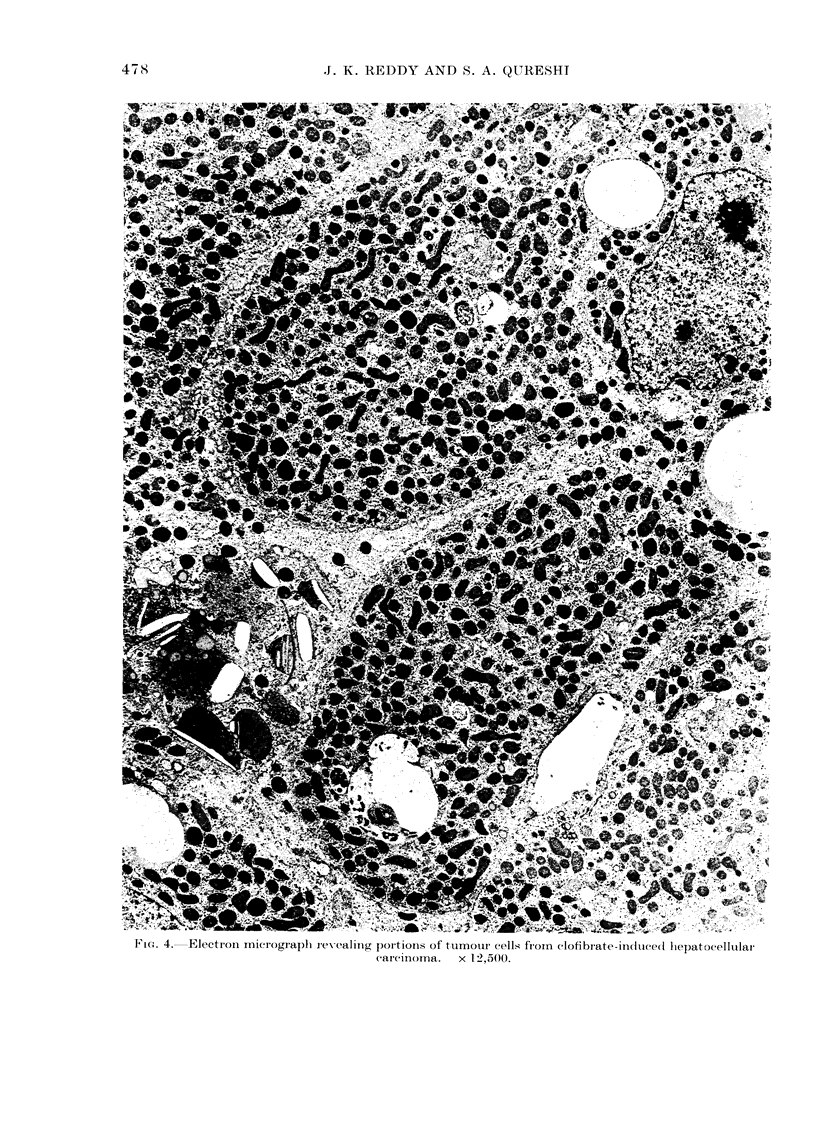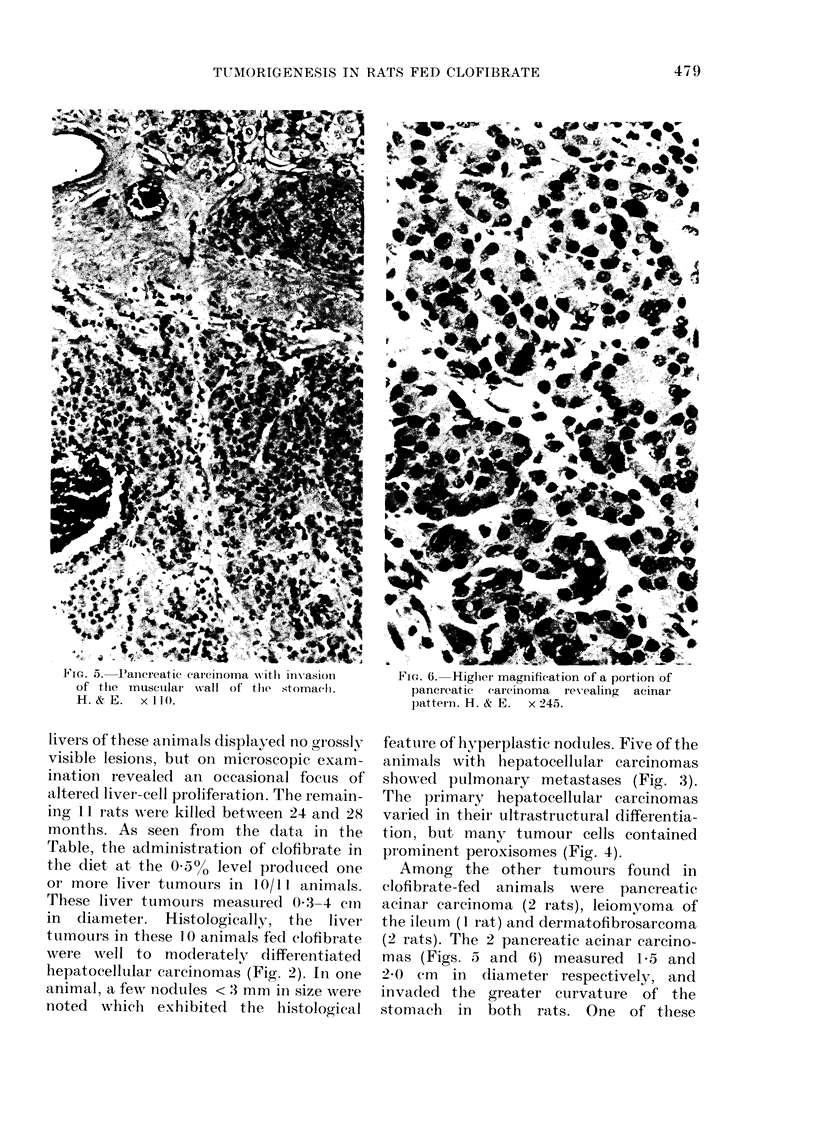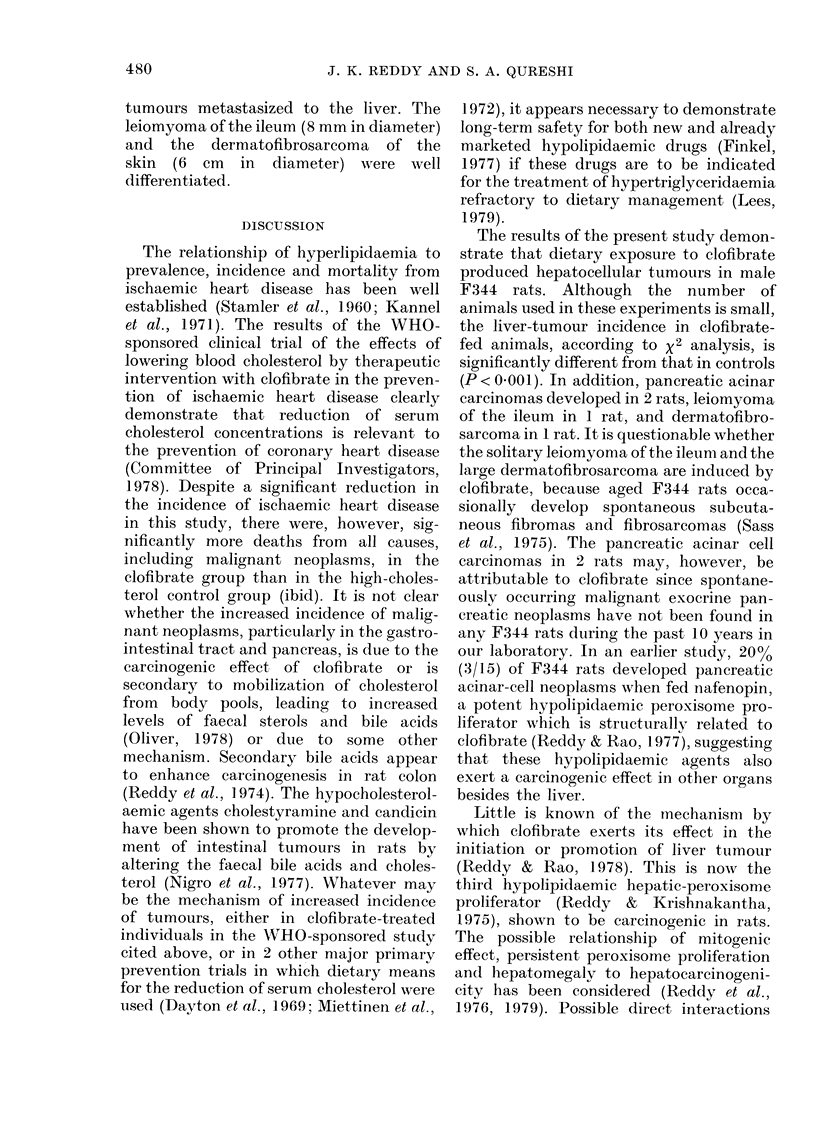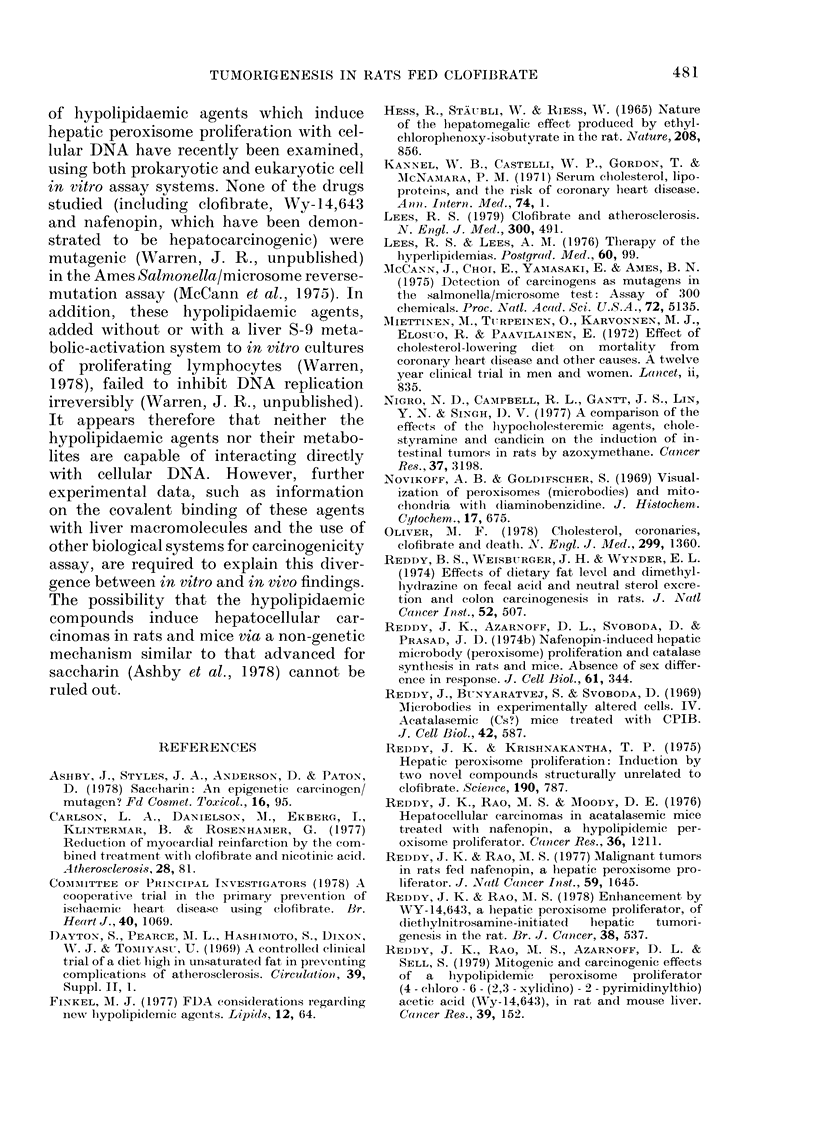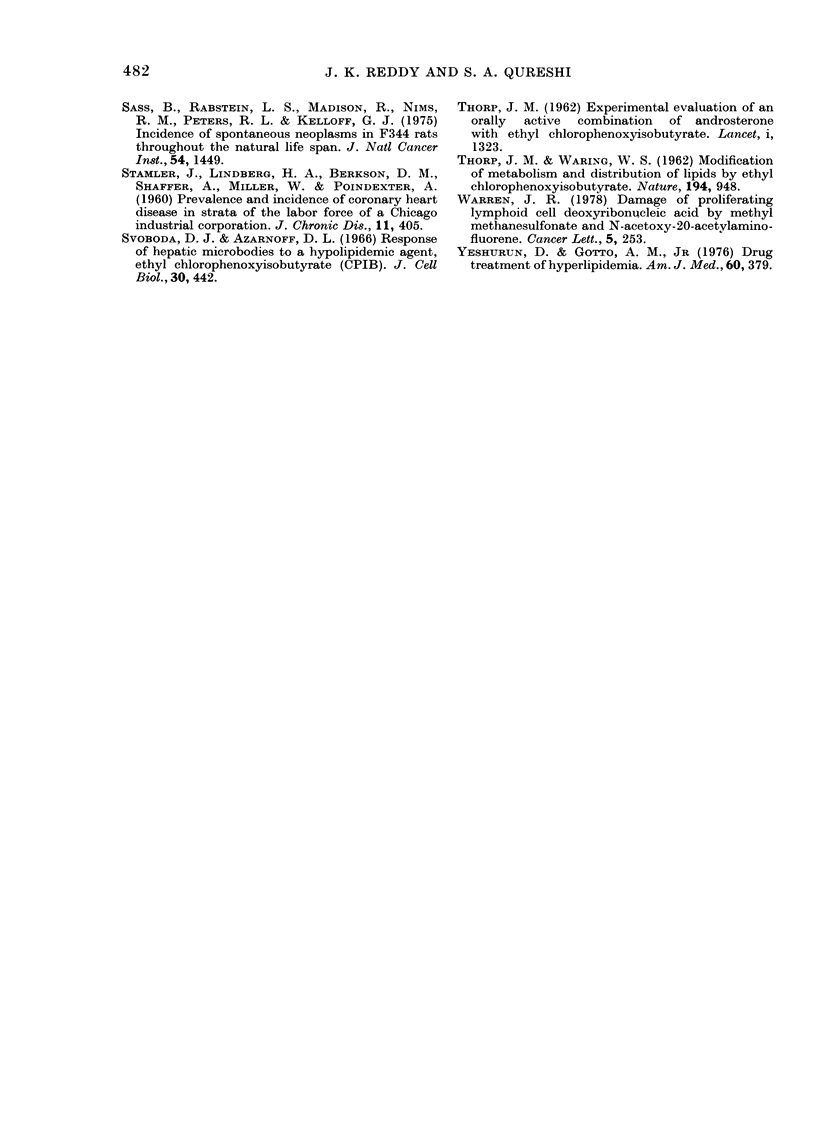# Tumorigenicity of the hypolipidaemic peroxisome proliferator ethyl-alpha-p-chlorophenoxyisobutyrate (clofibrate) in rats.

**DOI:** 10.1038/bjc.1979.203

**Published:** 1979-09

**Authors:** J. K. Reddy, S. A. Qureshi

## Abstract

**Images:**


*Br. J. Cancer* (1979) **40**, 476

### TUMORIGENICITY OF THE HYPOLIPIDAEMIC PEROXISOME PROLIFERATOR ETHYL-α-P-CHLOROPHENOXYISOBUTYRATE (CLOFIBRATE) IN RATS

J. K. REDDY AND S. A. QURESHI

*From the Department of Pathology, Northwestern University Medical School, Chicago, Illinois, U.S.A.*

Received 9 April 1979 Accepted 10 May 1979

**Summary.—Ethyl-α-p-chlorophenoxyisobutyrate (clofibrate), a hypolipidaemic drug which induces hepatomegaly and proliferation of peroxisomes in liver cells of rats and mice, was fed to 15 male F344 rats at a dietary concentration of 0·5% (v/w) for up to 28 months. Hepatocellular carcinomas developed in 10/11 (91%) rats killed between 24 and 28 months. Other tumours included carcinoma of the pancreas (2 rats), leiomyoma of the small intestine (1 rat) and a large dermatofibrosarcoma (1 rat). Clofibrate is the third hypolipidaemic peroxisome proliferator demonstrated to be hepatocarcinogenic in rats. These studies suggest that hypolipidaemic agents which are capable of producing a sustained hepatomegalic and peroxisome-proliferative effect also induce liver tumours.**

ETHYL α-p-chlorophenoxyisobutyrate (clofibrate, Fig. 1), which modifies lipid metabolism (Thorp, 1962; Thorp & Waring, 1962), is widely used in the treatment of hyperlipidaemia in an effort to prevent ischaemic heart disease (Yeshurun & Gotto, 1976; Lees & Lees, 1976; Carlson *et al.*, 1977). Clofibrate induces the proliferation of peroxisomes in liver cells in rats and mice, and this effect is sustained as long as the compound is administered (Hess *et al.*, 1965; Svoboda & Azarnoff, 1966; Reddy *et al.*, 1969). Since hypolipidaemic drugs such as clofibrate are being administered clinically for long periods (Finkel, 1977), it is essential to delineate the long-term biological effects of these agents. Previous studies from this laboratory have shown that nafenopin (2-methyl-2-[p-(1,2,3,4-tetrahydro-1-naphthyl) phenoxy] propionic acid = Su-13,437) and Wy-14,643 ([4-chloro-6-(2,3-xylidino)-2-pyrimidinylthio)-acetic acid), two structurally unrelated hypolipidaemic peroxisome proliferators, induce hepatocellular carcinomas in rats and mice (Reddy *et al.*, 1976, 1979; Reddy & Rao, 1977). We now report the development of tumours in male F344 rats treated chronically with clofibrate.

FIG. 1.—Structure of clofibrate (ethyl α-p-chlorophenoxyisobutyrate).

### MATERIALS AND METHODS

Male F344 rats, weighing 84–100 g, were obtained from ARS Sprague–Dawley, Madison, Wisconsin, and were housed in individual cages. Fifteen rats were fed clofibrate (Atromid-S®; Ayerst Laboratories, New York, N.Y.) at a concentration of 0·5% (v/w) in the ground rat chow for up to 28 months. Control rats were fed the same diet without the drug. Complete necropsies (with the exception of brain) were performed on all animals; tissues from selected organs were processed for light microscopy after fixing in neutral buffered formalin. Sections, 4–6 μm thick, were stained routinely with haematoxylin and eosin. The sections of the pancreatic tumours were also stained with mucicarmine, Fontana and Grimelius stains. For electron microscopy,

TABLE.—*Incidence of tumours in male F344 rats fed clofibrate*

| Group* | No. animals At start | No. animals Effective† | Animals with hepato-cellular carcinomas | Animals with additional tumours‡ Pancreatic acinar carcinoma | Leiomyoma intestine | Dermato-fibro-sarcoma |
|---|---|---|---|---|---|---|
| Clofibrate | 15 | 11 | 10 (91%§) | 2 | 1 | 1 |
| Control | 15 | 14 | 0 | 0 | 0 | 0 |

* Clofibrate was added to powdered chow at a level of 0·5% (v/w) and fed *ad libitum*. The control animals were fed the same chow without the drug, and include animals serving as long-term controls for chronic studies with other peroxisome proliferators.

† Number of animals killed between 24 and 28 months.

‡ In relation to the effective number. Significantly different ($P < 0{\cdot}001$) from animals fed control diet, as determined by $\chi^2$ test with 1 degree of freedom.

§ Occurring concurrently in animals with liver tumours.

samples of liver and liver tumours were fixed in cold 2·5% glutaraldehyde in 0·1M cacodylate buffer, pH 7·4, for 30–60 min and then post-fixed for 1 h in 1% $OsO_4$ in 0·1M *S*-collidine buffer, pH 7·4. After fixation, the tissues were dehydrated in ethanol and processed as described previously (Reddy *et al.*, 1974*b*). For cytochemical localization of peroxisome catalase, selected samples of liver and liver tumours were processed as described by Novikoff & Goldfischer (1969).

FIG. 2.—Hepatocellular carcinoma with trabecular pattern from a male rat fed clofibrate (0·5%) for 28 months. H. & E. ×85.

FIG. 3.—Hepatocellular carcinoma metastatic in lung. H. & E. ×112.

### RESULTS

Of the 15 rats fed clofibrate, 1 was killed at 13 months, and 3 more between 17 and 21 months. The markedly enlarged

FIG. 4.—Electron micrograph revealing portions of tumour cells from clofibrate-induced hepatocellular carcinoma. ×12,500.

FIG. 5.—Pancreatic carcinoma with invasion of the muscular wall of the stomach. H. & E. ×110.

FIG. 6.—Higher magnification of a portion of pancreatic carcinoma revealing acinar pattern. H. & E. ×245.

livers of these animals displayed no grossly visible lesions, but on microscopic examination revealed an occasional focus of altered liver-cell proliferation. The remaining 11 rats were killed between 24 and 28 months. As seen from the data in the Table, the administration of clofibrate in the diet at the 0·5% level produced one or more liver tumours in 10/11 animals. These liver tumours measured 0·3–4 cm in diameter. Histologically, the liver tumours in these 10 animals fed clofibrate were well to moderately differentiated hepatocellular carcinomas (Fig. 2). In one animal, a few nodules $< 3$ mm in size were noted which exhibited the histological feature of hyperplastic nodules. Five of the animals with hepatocellular carcinomas showed pulmonary metastases (Fig. 3). The primary hepatocellular carcinomas varied in their ultrastructural differentiation, but many tumour cells contained prominent peroxisomes (Fig. 4).

Among the other tumours found in clofibrate-fed animals were pancreatic acinar carcinoma (2 rats), leiomyoma of the ileum (1 rat) and dermatofibrosarcoma (2 rats). The 2 pancreatic acinar carcinomas (Figs. 5 and 6) measured 1·5 and 2·0 cm in diameter respectively, and invaded the greater curvature of the stomach in both rats. One of these tumours metastasized to the liver. The leiomyoma of the ileum (8 mm in diameter) and the dermatofibrosarcoma of the skin (6 cm in diameter) were well differentiated.

### DISCUSSION

The relationship of hyperlipidaemia to prevalence, incidence and mortality from ischaemic heart disease has been well established (Stamler *et al.*, 1960; Kannel *et al.*, 1971). The results of the WHO-sponsored clinical trial of the effects of lowering blood cholesterol by therapeutic intervention with clofibrate in the prevention of ischaemic heart disease clearly demonstrate that reduction of serum cholesterol concentrations is relevant to the prevention of coronary heart disease (Committee of Principal Investigators, 1978). Despite a significant reduction in the incidence of ischaemic heart disease in this study, there were, however, significantly more deaths from all causes, including malignant neoplasms, in the clofibrate group than in the high-cholesterol control group (ibid). It is not clear whether the increased incidence of malignant neoplasms, particularly in the gastrointestinal tract and pancreas, is due to the carcinogenic effect of clofibrate or is secondary to mobilization of cholesterol from body pools, leading to increased levels of faecal sterols and bile acids (Oliver, 1978) or due to some other mechanism. Secondary bile acids appear to enhance carcinogenesis in rat colon (Reddy *et al.*, 1974). The hypocholesterolaemic agents cholestyramine and candicin have been shown to promote the development of intestinal tumours in rats by altering the faecal bile acids and cholesterol (Nigro *et al.*, 1977). Whatever may be the mechanism of increased incidence of tumours, either in clofibrate-treated individuals in the WHO-sponsored study cited above, or in 2 other major primary prevention trials in which dietary means for the reduction of serum cholesterol were used (Dayton *et al.*, 1969; Miettinen *et al.*, 1972), it appears necessary to demonstrate long-term safety for both new and already marketed hypolipidaemic drugs (Finkel, 1977) if these drugs are to be indicated for the treatment of hypertriglyceridaemia refractory to dietary management (Lees, 1979).

The results of the present study demonstrate that dietary exposure to clofibrate produced hepatocellular tumours in male F344 rats. Although the number of animals used in these experiments is small, the liver-tumour incidence in clofibrate-fed animals, according to $\chi^2$ analysis, is significantly different from that in controls ($P < 0{\cdot}001$). In addition, pancreatic acinar carcinomas developed in 2 rats, leiomyoma of the ileum in 1 rat, and dermatofibrosarcoma in 1 rat. It is questionable whether the solitary leiomyoma of the ileum and the large dermatofibrosarcoma are induced by clofibrate, because aged F344 rats occasionally develop spontaneous subcutaneous fibromas and fibrosarcomas (Sass *et al.*, 1975). The pancreatic acinar cell carcinomas in 2 rats may, however, be attributable to clofibrate since spontaneously occurring malignant exocrine pancreatic neoplasms have not been found in any F344 rats during the past 10 years in our laboratory. In an earlier study, 20% (3/15) of F344 rats developed pancreatic acinar-cell neoplasms when fed nafenopin, a potent hypolipidaemic peroxisome proliferator which is structurally related to clofibrate (Reddy & Rao, 1977), suggesting that these hypolipidaemic agents also exert a carcinogenic effect in other organs besides the liver.

Little is known of the mechanism by which clofibrate exerts its effect in the initiation or promotion of liver tumour (Reddy & Rao, 1978). This is now the third hypolipidaemic hepatic-peroxisome proliferator (Reddy & Krishnakantha, 1975), shown to be carcinogenic in rats. The possible relationship of mitogenic effect, persistent peroxisome proliferation and hepatomegaly to hepatocarcinogenicity has been considered (Reddy *et al.*, 1976, 1979). Possible direct interactions of hypolipidaemic agents which induce hepatic peroxisome proliferation with cellular DNA have recently been examined, using both prokaryotic and eukaryotic cell *in vitro* assay systems. None of the drugs studied (including clofibrate, Wy-14,643 and nafenopin, which have been demonstrated to be hepatocarcinogenic) were mutagenic (Warren, J. R., unpublished) in the Ames *Salmonella*/microsome reverse-mutation assay (McCann *et al.*, 1975). In addition, these hypolipidaemic agents, added without or with a liver S-9 metabolic-activation system to *in vitro* cultures of proliferating lymphocytes (Warren, 1978), failed to inhibit DNA replication irreversibly (Warren, J. R., unpublished). It appears therefore that neither the hypolipidaemic agents nor their metabolites are capable of interacting directly with cellular DNA. However, further experimental data, such as information on the covalent binding of these agents with liver macromolecules and the use of other biological systems for carcinogenicity assay, are required to explain this divergence between *in vitro* and *in vivo* findings. The possibility that the hypolipidaemic compounds induce hepatocellular carcinomas in rats and mice *via* a non-genetic mechanism similar to that advanced for saccharin (Ashby *et al.*, 1978) cannot be ruled out.

### REFERENCES

Ashby, J., Styles, J. A., Anderson, D. & Paton, D. (1978) Saccharin: An epigenetic carcinogen/mutagen? *Fd Cosmet. Toxicol.*, **16**, 95.

Carlson, L. A., Danielson, M., Ekberg, I., Klintermar, B. & Rosenhamer, G. (1977) Reduction of myocardial reinfarction by the combined treatment with clofibrate and nicotinic acid. *Atherosclerosis*, **28**, 81.

Committee of Principal Investigators (1978) A cooperative trial in the primary prevention of ischaemic heart disease using clofibrate. *Br. Heart J.*, **40**, 1069.

Dayton, S., Pearce, M. L., Hashimoto, S., Dixon, W. J. & Tomiyasu, U. (1969) A controlled clinical trial of a diet high in unsaturated fat in preventing complications of atherosclerosis. *Circulation*, **39**, Suppl. II, 1.

Finkel, M. J. (1977) FDA considerations regarding new hypolipidemic agents. *Lipids*, **12**, 64.

Hess, R., Stäubli, W. & Riess, W. (1965) Nature of the hepatomegalic effect produced by ethyl-chlorophenoxy-isobutyrate in the rat. *Nature*, **208**, 856.

Kannel, W. B., Castelli, W. P., Gordon, T. & McNamara, P. M. (1971) Serum cholesterol, lipoproteins, and the risk of coronary heart disease. *Ann. Intern. Med.*, **74**, 1.

Lees, R. S. (1979) Clofibrate and atherosclerosis. *N. Engl. J. Med.*, **300**, 491.

Lees, R. S. & Lees, A. M. (1976) Therapy of the hyperlipidemias. *Postgrad. Med.*, **60**, 99.

McCann, J., Choi, E., Yamasaki, E. & Ames, B. N. (1975) Detection of carcinogens as mutagens in the salmonella/microsome test: Assay of 300 chemicals. *Proc. Natl. Acad. Sci. U.S.A.*, **72**, 5135.

Miettinen, M., Turpeinen, O., Karvonnen, M. J., Elosuo, R. & Paavilainen, E. (1972) Effect of cholesterol-lowering diet on mortality from coronary heart disease and other causes. A twelve year clinical trial in men and women. *Lancet*, ii, 835.

Nigro, N. D., Campbell, R. L., Gantt, J. S., Lin, Y. N. & Singh, D. V. (1977) A comparison of the effects of the hypocholesteremic agents, cholestyramine and candicin on the induction of intestinal tumors in rats by azoxymethane. *Cancer Res.*, **37**, 3198.

Novikoff, A. B. & Goldifscher, S. (1969) Visualization of peroxisomes (microbodies) and mitochondria with diaminobenzidine. *J. Histochem. Cytochem.*, **17**, 675.

Oliver, M. F. (1978) Cholesterol, coronaries, clofibrate and death. *N. Engl. J. Med.*, **299**, 1360.

Reddy, B. S., Weisburger, J. H. & Wynder, E. L. (1974) Effects of dietary fat level and dimethylhydrazine on fecal acid and neutral sterol excretion and colon carcinogenesis in rats. *J. Natl Cancer Inst.*, **52**, 507.

Reddy, J. K., Azarnoff, D. L., Svoboda, D. & Prasad, J. D. (1974b) Nafenopin-induced hepatic microbody (peroxisome) proliferation and catalase synthesis in rats and mice. Absence of sex difference in response. *J. Cell Biol.*, **61**, 344.

Reddy, J., Bunyaratvej, S. & Svoboda, D. (1969) Microbodies in experimentally altered cells. IV. Acatalasemic (Cs?) mice treated with CPIB. *J. Cell Biol.*, **42**, 587.

Reddy, J. K. & Krishnakantha, T. P. (1975) Hepatic peroxisome proliferation: Induction by two novel compounds structurally unrelated to clofibrate. *Science*, **190**, 787.

Reddy, J. K., Rao, M. S. & Moody, D. E. (1976) Hepatocellular carcinomas in acatalasemic mice treated with nafenopin, a hypolipidemic peroxisome proliferator. *Cancer Res.*, **36**, 1211.

Reddy, J. K. & Rao, M. S. (1977) Malignant tumors in rats fed nafenopin, a hepatic peroxisome proliferator. *J. Natl Cancer Inst.*, **59**, 1645.

Reddy, J. K. & Rao, M. S. (1978) Enhancement by WY-14,643, a hepatic peroxisome proliferator, of diethylnitrosamine-initiated hepatic tumorigenesis in the rat. *Br. J. Cancer*, **38**, 537.

Reddy, J. K., Rao, M. S., Azarnoff, D. L. & Sell, S. (1979) Mitogenic and carcinogenic effects of a hypolipidemic peroxisome proliferator (4 - chloro - 6 - (2,3 - xylidino) - 2 - pyrimidinylthio) acetic acid (Wy-14,643), in rat and mouse liver. *Cancer Res.*, **39**, 152.

SASS, B., RABSTEIN, L. S., MADISON, R., NIMS, R. M., PETERS, R. L. & KELLOFF, G. J. (1975) Incidence of spontaneous neoplasms in F344 rats throughout the natural life span. *J. Natl Cancer Inst.*, **54**, 1449.

STAMLER, J., LINDBERG, H. A., BERKSON, D. M., SHAFFER, A., MILLER, W. & POINDEXTER, A. (1960) Prevalence and incidence of coronary heart disease in strata of the labor force of a Chicago industrial corporation. *J. Chronic Dis.*, **11**, 405.

SVOBODA, D. J. & AZARNOFF, D. L. (1966) Response of hepatic microbodies to a hypolipidemic agent, ethyl chlorophenoxyisobutyrate (CPIB). *J. Cell Biol.*, **30**, 442.

THORP, J. M. (1962) Experimental evaluation of an orally active combination of androsterone with ethyl chlorophenoxyisobutyrate. *Lancet*, i, 1323.

THORP, J. M. & WARING, W. S. (1962) Modification of metabolism and distribution of lipids by ethyl chlorophenoxyisobutyrate. *Nature*, **194**, 948.

WARREN, J. R. (1978) Damage of proliferating lymphoid cell deoxyribonucleic acid by methyl methanesulfonate and N-acetoxy-20-acetylamino-fluorene. *Cancer Lett.*, **5**, 253.

YESHURUN, D. & GOTTO, A. M., JR (1976) Drug treatment of hyperlipidemia. *Am. J. Med.*, **60**, 379.